# Distributed Neural Processing Predictors of Multi-dimensional Properties of Affect

**DOI:** 10.3389/fnhum.2017.00459

**Published:** 2017-09-14

**Authors:** Keith A. Bush, Cory S. Inman, Stephan Hamann, Clinton D. Kilts, G. Andrew James

**Affiliations:** ^1^Brain Imaging Research Center, University of Arkansas for Medical Sciences, Little Rock AR, United States; ^2^Department of Psychology, Emory University, Atlanta GA, United States

**Keywords:** affect, IAPS, MVPA, SVM, fMRI, neural representation, neuromodulation

## Abstract

Recent evidence suggests that emotions have a distributed neural representation, which has significant implications for our understanding of the mechanisms underlying emotion regulation and dysregulation as well as the potential targets available for neuromodulation-based emotion therapeutics. This work adds to this evidence by testing the distribution of neural representations underlying the affective dimensions of valence and arousal using representational models that vary in both the degree and the nature of their distribution. We used multi-voxel pattern classification (MVPC) to identify whole-brain patterns of functional magnetic resonance imaging (fMRI)-derived neural activations that reliably predicted dimensional properties of affect (valence and arousal) for visual stimuli viewed by a normative sample (*n* = 32) of demographically diverse, healthy adults. Inter-subject leave-one-out cross-validation showed whole-brain MVPC significantly predicted (*p* < 0.001) binarized normative ratings of valence (positive vs. negative, 59% accuracy) and arousal (high vs. low, 56% accuracy). We also conducted group-level univariate general linear modeling (GLM) analyses to identify brain regions whose response significantly differed for the contrasts of positive versus negative valence or high versus low arousal. Multivoxel pattern classifiers using voxels drawn from all identified regions of interest (all-ROIs) exhibited mixed performance; arousal was predicted significantly better than chance but worse than the whole-brain classifier, whereas valence was not predicted significantly better than chance. Multivoxel classifiers derived using individual ROIs generally performed no better than chance. Although performance of the all-ROI classifier improved with larger ROIs (generated by relaxing the clustering threshold), performance was still poorer than the whole-brain classifier. These findings support a highly distributed model of neural processing for the affective dimensions of valence and arousal. Finally, joint error analyses of the MVPC hyperplanes encoding valence and arousal identified regions within the dimensional affect space where multivoxel classifiers exhibited the greatest difficulty encoding brain states – specifically, stimuli of moderate arousal and high or low valence. In conclusion, we highlight new directions for characterizing affective processing for mechanistic and therapeutic applications in affective neuroscience.

## Introduction

The ability to rapidly and accurately interpret multiple dimensions of emotion information in affective stimuli is essential to social behavior and self-preservation. Numerous studies have sought to elucidate the neural mechanisms by which we process complex sensory information to arrive at affective cognitions. Functional magnetic resonance imaging (fMRI) studies have uniquely informed the neurobiology of human emotion and affective processing (Bush et al., 2000; Killgore and Yurgelun-Todd, 2007; Gerber et al., 2008; Wager et al., 2008; Hagan et al., 2009; Posner et al., 2009; Colibazzi et al., 2010). Indeed, there is growing evidence to suggest that emotion processing is broadly encoded by generalized networks that are distributed throughout the brain (Baucom et al., 2012; Lindquist et al., 2012; Chang et al., 2015; Saarimäki et al., 2016).

This knowledge has significant implications for our understanding of the mechanisms underlying emotion regulation and dysregulation as well as the potential targets available for neuromodulation-based emotion therapeutics. Several recent studies have explored neurofeedback-guided volitional control of specific anatomical regions, e.g., amygdala, based on their presumed superordinate role in encoding both emotional valence and arousal (Johnston et al., 2010; Zotev et al., 2013; Paret et al., 2014; Young et al., 2014). However, as our understanding of emotion as a distributed cognitive process becomes more concrete, the assumptions underlying region-specific emotion regulation approaches may need to be revised.

Initial hypotheses of the neural basis of emotion relied on assumptions drawn from two influential theoretical models: the discrete emotion view and the dimensional affect view (Hamann, 2012). Discrete emotion theory proposes the existence of a small set of emotional primitives (basic emotions, e.g., happiness, sadness, anger, fear, disgust, and surprise) each having unique causal factors, physiological responses, and developmental onsets among other properties (Ekman, 1999). Dimensional affect theory proposes that combinations of graded fundamental properties, namely valence (the degree of pleasantness or lack thereof), arousal (the degree of intensity), and possibly others (e.g., dominance), are shaped by other cognitive processes (e.g., attribution and social context) to form emotions (Lindquist et al., 2012). These theoretical models are important because they originally implied differing neurobiological origins of affective processing which could be empirically tested (Scaratino and Griffiths, 2011). Basic emotions are typically proposed to be mediated by a set of evolutionarily determined, specific neuroanatomical substrates that are dedicated to each basic emotion (Ekman, 1999; Izard, 2011) whereas dimensional affect theories typically posit that emotions arise from cooperation among multiple, distributed neural processing networks (Lindquist et al., 2012).

Recent meta-analyses suggest that approaches to understanding the neural correlates of emotions that rely on simple one-to-one mappings between emotion constructs and individual brain regions are ultimately insufficient, and that more complex relationships, such as distributed functional networks, are required (Vytal and Hamann, 2010; Lindquist et al., 2012). Multivoxel (or multivariate) pattern analysis (MVPA) represents a promising approach to detecting highly distributed activation patterns corresponding to emotions (Hamann, 2012). Within the context of functional neuroimaging datasets, MVPA elaborates cognitive states as functions of coordinated activity among ensembles of multiple and distributed brain voxels (Haxby et al., 2001). MVPA addresses the limitations of the univariate approach (Habeck and Stern, 2010) and has been shown to offer superior predictive performance over univariate analysis (Haynes and Rees, 2006; Norman et al., 2006; O’Toole et al., 2007).

MVPA applications in affective neuroimaging have identified novel functional anatomical models of known emotional states as neural responses to variation in stimulus properties, e.g., modality and intensity independence (Peelen et al., 2010), modality-dependent voice-activation (Ethofer et al., 2009), dynamic facial expression (Said et al., 2010), facial expression of fear (Pessoa and Padmala, 2007), and imagined or recalled emotional situations (Sitaram et al., 2011; Kassam et al., 2013). In these works, MVPA was used either to disambiguate the possible neural encoding roles of a theory-driven univariately defined brain region-of-interest (ROI) or to confirm the roles of known ROIs.

MVPA-based methods have also been applied to broadly characterize representations of emotion processing. Indeed, multivoxel pattern classification (MVPC), a form of MVPA, has been used to classify the dimensional affective properties of pictures using low-dimensional, distributed neural features (Baucom et al., 2012) as well as to identify neural activation patterns that predict affective responses but are not necessarily constrained to anatomical regions implicated by univariate analyses (Chang et al., 2015). A recent, influential MVPC-based analysis of discrete emotions (Saarimäki et al., 2016) identified overlapping and distributed neural patterns of the six basic emotions (primarily) along the medial prefrontal and medial posterior regions. Combined, these MVPC-based findings suggest a distributed neural basis underlies both the discrete and dimensional theoretical models of emotion (Kragel and LaBar, 2016).

In the current study, our goal was to extend the existing affective neuroimaging literature regarding the neural bases of dimensional affect in four important ways. First, we used whole-brain MVPC to map the neural distribution of regions encoding the affective dimensions of valence and arousal for visual stimuli within a normative sample (*n* = 32) of demographically diverse (across sex, race, age, and education) subjects. Second, using univariate analysis to derive ROIs canonically associated with affective processing, we applied MVPC to understand the ROIs’ individual and collective predictive performance in characterizing dimensional affective properties compared against the predictive performance of the whole-brain classifier. In a trade-off, this approach sacrifices the theoretically optimal performance MVPC can achieve through voxel-wise feature selection methods (e.g., Baucom et al., 2012) in order to gain deeper insights into the processing roles made by discrete brain regions canonically associated with dimensional affective processing. Third, we tasked MVPC with classification of experimental stimuli sampled continuously from the affective dimensions of valence and arousal, a more plausible test of discrimination of these dimensions compared to image stimuli chosen to cluster at the dimensional extremes (Baucom et al., 2012). Fourth, we sought to exploit the mathematical structure of our MVPC algorithm by projecting its multivoxel decision surface into both anatomical and affect space to better understand the distribution of neural processing patterns that arise from presenting multi-dimensional affective stimuli to a relatively large, normative sample of subjects. This work would not only generate a more nuanced view of the neural processing models underlying emotion and affect, but may also inform methodological approaches to regulating emotion via intrinsic or extrinsic neuromodulation interventions. Consistent with prior studies, we focused on behavioral and fMRI responses to items in the International Affective Picture System (IAPS), a well-characterized stimulus set often used in the study of affect processing that captures the richness of multi-dimensional information encoded by affective stimuli.

## Materials and Methods

### Study Overview

The project conducted retrospective analyses of data acquired from the Encoding of Affective Pictures task (see below) of the Cognitive Connectome project, a comprehensive exploration of normative variance in the neural encoding of behavior and cognition described in detail elsewhere (Gess et al., 2014). All study procedures were conducted in the Brain Imaging Research Center (BIRC) at the University of Arkansas for Medical Sciences (UAMS). Study participation was typically conducted in two sessions on separate days. Session 1 included obtaining written informed consent, determining if participants met exclusionary criteria via structured clinical interview (SCID-I/NP), administering behavioral surveys and questionnaires (such as the State-Trait Anxiety Inventory and Big Five Personality Inventory), and the first of 2 h-long neuroimaging sessions (with neuroimaging session order counterbalanced across participants). Session 2 included comprehensive neuropsychological assessment (lasting 3–4 h) and the second neuroimaging session. All subjects gave written informed consent and all procedures were conducted with approval and oversight by the UAMS Institutional Review Board.

### Participants

Thirty-two participants completed the Encoding of Affective Pictures task of the Cognitive Connectome project. The participant sample had the following demographic characteristics: age [mean(SD)]: 29.7(9.3), range 19–50; sex: 16 (50%) female; race/ethnicity: 20 (63%) self-reporting as White or Caucasian, 10 (31%) as Black or African–American, 2 (6%) as Hispanic or Latino; education [mean(SD)]: 15.4(2.2) years, range 10–19.

### Stimuli

The stimulus set consisted of 90 color images depicting a broad range of emotional content (e.g., aggression, accidents, injury, social scenes, inanimate objects). Fifty three images were selected from the IAPS (Lang et al., 2008) and 37 images from a corpus of affective images developed by the Hamann Cognitive Neuroscience Lab (HCNL) (see Supplemental Table 1). The 53 IAPS stimuli included normative ratings of valence (V), arousal (A) and dominance; additionally, an independent sample (*n* = 6) of participants rated all 90 stimuli for valence, arousal, and image complexity. The 90 images consisted of 30 positive (high valence), 30 neutral (moderate valence), and 30 negative (low valence) images. The stimulus set was subdivided into two independent sets of 45 images (Sets A and B), with 15 positive, 15 neutral, and 15 negative images each.

### Task

Images from Set A or B (counterbalanced across participants) were presented to the participant as an incidental memory task, the “Encoding of Affective Pictures” task. Participants were asked to rate each image valence as emotionally negative, neutral, or positive by a button press response with their right index, middle, or ring finger (respectively) using an 8-button bimanual response pad (HHSC-2x4-C, Current Designs, Inc., Philadelphia, PA, United States). Each image was presented for 2.5 s with a 2–6 s intertrial interval (3.88 s mean ITI) using a pre-determined pseudo-randomized order optimized for event-related fMRI with the optseq2 program available through FreeSurfer (Greve, 2006). Total task duration was 5 min 14 s. Participants then underwent three distractor fMRI tasks (verbal fluency, visuospatial judgment, and *n*-back working memory tasks) followed by a forced-choice recognition memory task using all 90 images from Sets A and B. This work focuses only on the data from the Encoding of Affective Pictures task.

### Affect Label Scaling

The 53 IAPS images had normative ratings of valence and arousal based upon a 9-point Likert scale, whereas an independent sample (*n* = 6) rated all 90 images for valence and arousal using a 5-point Likert scale. To remove possible latent linear bias in the ratings of the smaller independent sample, mean affect ratings for the 37 HCNL images were re-scaled to the normative 9-point Likert scale as follows. The 53 IAPS images (having both normative and independent ratings of valence and arousal) were used as a training dataset. Iteratively reweighted least squares regression (robustfit) was used to predict normative (IAPS) ratings from independent (HCNL) ratings separately for both valence and arousal. Predicted IAPS ratings for the remaining 37 HCNL images were highly significant for valence (ρ = 0.924, *p* < 0.001) and arousal (ρ = 0.624, *p* < 0.001), supporting this scaling approach. Henceforth, the combined (true and predicted) IAPS ratings for all 90 images are referred to as the IAPS ratings (see Supplementary Table 1 for individual stimulus ratings and identifiers).

### Image Acquisition

Imaging data were acquired using a Philips 3T Achieva X-series MRI scanner (Philips Healthcare, Eindhoven, The Netherlands). Anatomic images were acquired with a MPRAGE sequence [matrix = 256 × 256, 220 sagittal slices, TR/TE/FA = shortest/shortest/8°, final resolution = 0.94 mm × 0.94 mm × 1 mm. For functional images, initial participants (*n* = 18)] were acquired using an eight-channel head coil with an echo planar imaging (EPI) sequence (TR/TE/FA = 2000 ms/30 ms/90°, FOV = 240 × 240 mm, matrix = 80 × 80, 37 oblique slices parallel to orbitofrontal cortex to reduce sinus artifact, interleaved ascending slice acquisition, slice thickness = 4 mm, final resolution 3.0 × 3.0 × 4.0). Functional images for later participants (*n* = 14) were acquired using a 32-channel head coil with the following EPI sequence parameters: TR/TE/FA = 2000 ms/30 ms/90°, FOV = 240 × 240 mm, matrix = 80 × 80, 37 oblique slices, ascending sequential slice acquisition, slice thickness = 2.5 mm with 0.5 mm gap, final resolution 3.0 mm × 3.0 mm × 3.0 mm. Parameters for the 32-channel coil were selected to reduce orbitofrontal signal loss due to sinus artifact.

We explicitly tested if head coil influenced task-related neural pattern classification performance by conducting a two-sample, two-tailed *t*-test for group differences in MVPC prediction accuracy (eight-channel vs. 32-channel coil) for positive vs. negative valence, high vs. low arousal, and positive vs. negative self-reported valence (contrast details described below). No significant group differences were found. Given the lack of significant differences in our univariate contrasts, head coil was not included as a covariate of interest for the remainder of the analyses.

### Image Preprocessing

All MRI data preprocessing was conducted in AFNI (Version AFNI_16.1.07) (Cox, 1996) unless otherwise noted. Anatomic data underwent skull stripping, spatial normalization to the icbm452 brain atlas, and segmentation into white matter (WM), gray matter (GM), and cerebrospinal fluid (CSF) with FSL (Jenkinson et al., 2012). Functional data underwent despiking; slice correction; deobliquing (to 3 mm × 3 mm × 3 mm voxels); motion correction (using the 10th timepoint); transformation to the spatially normalized anatomic image; regression of mean timecourse of WM voxels, mean timecourse of CSF voxels, and 24 motion parameters (Power et al., 2014, 2015); spatial smoothing with a 6-mm FWHM Gaussian kernel; and, scaling to percent signal change. fMRI scans with head motion exceeding 3 mm lateral movement were excluded from subsequent analyses.

### Gray Matter (GM) Masking

Group-level GM masks were constructed by voxel-wise thresholding over sets of participants’ GM segmentation masks using an inclusion threshold of 50% – i.e., only voxels identified as gray matter for at least half of participants using to form the mask were included. GM masks were created using sets of participants including all but one (*n* = 31) to conduct unbiased MVPC cross-validation. These masks were tested against the standard Talairach atlas for inclusion of subcortical structures. To ensure the inclusion of important subcortical structures in MVPA that may be lost during preprocessing, we computed the percent of voxels (combined left and right) kept on average in these GM masks for the caudate (63.2%), putamen (37.3%), and thalamus (16.8%).

### Univariate Analyses (GLM)

Group-level univariate analyses were conducted as follows. First, image stimuli were binarized with respect to their targeted affective properties. Images were classified as having either a positive or negative valence according to their normative ratings (positive valence or V_pos_ if V ≥ 5.0; otherwise negative valence or V_neg_). Similarly, each image was classified as having either high or low arousal on the basis of normative arousal rating (high arousal or A_high_ if A ≥ 5.0; otherwise low arousal or A_low_). Our selection of this binarization approach was motivated by the lack of a consensus definition for “cut-off” criteria separating neutral stimuli from positive and negative stimuli (see the Discussion for alternative label segmentations). Each participant’s GM-masked voxel timeseries underwent event-related general linear modeling (GLM) using AFNI’s 3dDeconvolve and 3dREMLfit programs with two predictors: valence modeling used V_pos_ and V_neg_; arousal modeling used A_high_ and A_low_. Contrast activation maps were also created for each participant for both valence, (V_pos_ – V_neg_), and arousal, (A_high_ – A_low_), respectively.

### Cluster Analysis

Group-level cluster masks were constructed as follows. Group contrast activation maps were calculated voxel-wise via one sample *t*-tests across participants. These maps were thresholded at an uncorrected *p* ≤ 0.001 (corresponding to two-tailed |t| ≥ 3.63 for df = 31). We executed AFNI’s 3dFWHMx function using the ‘acf’ option on the residual maps resulting from each participant’s univariate GLM, yielding three estimated smoothness parameters per participant. We then executed AFNI’s 3dClustSim function using the ‘acf’ option, supplying both the median values of the estimated smoothness parameters as well as the GM mask of the appropriate participant set as inputs, yielding the minimum cluster sizes allowable at a corrected significance of *p* ≤ 0.05 (two-sided thresholding, NN = 1), which were applied to the thresholded group activation maps to generate the group cluster maps. The minimum cluster size over all subjects was computed to be 12.8 voxels.

### Cluster Stability Analysis

Group-level cluster masks were compared against cross-validated cluster masks (i.e., *n* = 31 group-level cluster masks) to determine the stability of the cluster masks. For each subject, we conducted the following stability analysis. Each ROI of the subject’s cross-validated cluster mask (valence and arousal, respectively), in order descending from the largest cluster to the smallest cluster (by voxel size), was iteratively assigned to a group-level cluster mask ROI by prioritizing the smallest Euclidean distance between the clusters’ center of masses. Each group-level cluster was assigned once per subject. For each of the group-level clusters, we then calculated the fraction of subjects having a matching cluster as well as group-wise mean and standard deviations over matched cluster size and matched cluster distance.

### Univariate Analyses (Beta-Series)

The beta-series method (Rissman et al., 2004) and MVPC (which relies on beta-series to form the feature space) both assume low trial-to-trial variance – specifically, that greater variance exists between trials of different categories than within trials of the same category (Abdulrahman and Henson, 2016). In other words, differences between the beta-series method and the canonical univariate GLM approach could pose a confound to predictions inferred via MVPC. We tested this assumption by conducting univariate analysis using the beta-series method and contrasting these results to the GLM approach. Beta-series were extracted using the -stim_times_IM flag of 3dDeconvolve to construct the model which we subsequently solved via 3dLSS. We refer to these stimulus-driven activation maps as brain states for the remainder of this work. The resulting activation maps β[subject, stim], paired with the appropriate IAPS labels, form a dataset of tuples {β[subject, stim], V[stim], A[stim]} for use in subsequent analysis. We address trial-to-trial variance by voxelwise correlation of univariate beta-series activations with univariate GLM activations for both valence and arousal contrasts.

### Multivariate (i.e., Multivoxel) Pattern Classification

MVPC was implemented using linear support vector machine (SVM) classification (Boser et al., 1992) using the default implementation found within the Matlab Statistics Toolbox (The Mathworks, 2015).

### Whole-Brain Variants of MVPC

To investigate the predictive information contained within anatomically unrestricted fMRI, we performed linear SVM classification on GM-masked whole-brain fMRI brain imaging state values. GM masks for each participant were computed by applying the GM threshold to the (*n* = 31) other participants of the study, i.e., according to an inter-subject cross-validation. However, these GM masks were used for both inter- and intra-subject whole-brain classifications.

### ROI-Based Variants of MVPC

We performed several ROI-based predictions to investigate the anatomical loci of affective processing. These predictions were performed on GM-masked whole-brain fMRI brain imaging state values that were further masked according to the desired anatomical configuration. We compared three mask variants: all ROIs (aROI), all ROIs with relaxed masking criteria (rcaROI), and individual ROIs (iROI). The aROI mask was comprised of all surviving univariate clusters for the desired classification task (either valence or arousal). The rcaROI {params} mask was comprised of all surviving clusters when thresholding has been relaxed according to a set of specific relaxation criteria denoted by parameters {params}. The iROI mask was comprised of a single ROI cluster. Cross-validation, training, prediction, and statistical analysis of these MVPC variants were conducted identically to whole-brain.

### Inter-subject Cross-Validation

Leave-one-out-cross-validation (LOOCV) was performed subject-wise (number of total subjects = N_subj_). Thus, N_subj_-1 subjects’ data were used to predict the data of the remaining subject. For each of the N_subj_ test subjects, the training and testing set was formed as follows. The test subject’s stimuli and brain states were divided into two classes (positive) L+ and (negative) L- with respect to the classification being performed. The larger set of classes was identified (size = N_max_) and set as the rotate_set. The smaller set (size = N_min_) is set to the static_set. For N_max_ rotations the first N_min_ stimuli from the rotate_set are selected and combined with the static_set creating the test dataset having equal numbers of L+ and L-. The corresponding stimuli from all training subjects are extracted to form the training dataset. This rotation and extraction process ensures that all test/train combinations for each test subject are evaluated and that the training and testing datasets are formed from similar stimuli. Therefore, the independent variation in this process is the uniqueness of the test subject’s brain states compared to that of the training group. A linear SVM classifier was then fit to the training set and tested on the test set producing a set of hyperplane distances whose sign indicated the predicted class. Test set classes and predicted hyperplanes were saved for succeeding statistical analysis [accuracy, confidence interval (CI), *t*-score, true positive rate (TPR), false positive rate (FPR)]. For each test subject, N_max_ accuracies are averaged to produce the accuracy of that single fold of LOOCV. The resulting N_subj_ accuracies formed the LOOCV distribution of inter-subject prediction performance.

### Intra-subject Cross-Validation

Cross-validation was performed stimulus-wise within each subject. Each subject’s stimuli and brain states were divided into two sets (positive classes) L+ and (negative classes) L- with respect to the classification being performed. The larger set of classes was identified (size = N_max_) and set as the rotate_set. The smaller set (size = N_min_) is set to the static_set. For N_max_ rotations the first N_min_ stimuli from the rotate_set are selected and combined with the static_set creating the test dataset having equal numbers of L+ and L-. On this combined dataset, normal LOOCV was performed stimulus-wise and accuracy was computed. The N_max_ accuracies were averaged to produce the accuracy of a single subject. The resulting N_subj_ accuracies formed the distribution of the intra-subject prediction performance.

### Gaussian Process Model of Misclassification Accuracy

A Gaussian Process Regression Model (GPRM) (Rasmussen and Williams, 2006), i.e., kriging model, was used to model the mean joint affect misclassification accuracy of the linear SVM model. For each stimulus image, the mean joint misclassification accuracy of that stimulus was computed as follows. Mean misclassification accuracy for each stimulus was computed independently across all subjects separately for valence and arousal. These accuracies were assumed independent and the mean joint misclassification accuracy was formed by the product of the mean valence and mean arousal misclassification accuracies across subjects. We warped these probabilities to a continuous range by projection using the hyperbolic tangent: y = atanh(2^∗^p-1). A GPRM was then fit to y as a function of the mean valence and arousal Likert scores for these images ({V,A} = x). We used the default implementation of GPRM found within the Matlab Statistics Toolbox (The Mathworks, 2015). Estimates of y over ranges V = [1.0,9.0], A = [2.0,7.5] were then predicted using the model and projected back into probability space using the projection p = (tanh(y)+1)/2.

### Experiment Design

To meet our objectives of mapping the neural distribution of regions encoding the affective dimensions of valence and arousal for visual stimuli, as well as measure the predictive performance of these encodings, we conducted both inter-subject and intra-subject whole-brain gray matter (whole-brain) MVPC experiments independently for both valence and arousal ratings of stimuli. Inter-subject classification predicted dimensional affect ratings using the brain states of independent subjects that were not part of the classifier’s training data. Intra-subject classification predicted dimensional affect ratings using the brain states evoked by stimuli (within a single subject) that were not used for training. Classifier training and testing followed similar courses within these two models, changing only with respect to the quantity of the training and testing data used and model-specific technical details regarding un-biased cross-validation and performance analysis (see inter- versus intra-subject cross-validation methods).

To compare and contrast the fidelity of ROIs as predictors of dimensional affect ratings, either as discrete predictors or as an intact multiple-ROI predictor to inform our understanding of the functional role that specific anatomical regions play in dimensional affective processing, we conducted inter-subject MVPC experiments independently for both valence and arousal ratings of stimuli (we conducted only whole-brain analysis for self-reported valence), using the following set of multi-voxel patterns as input:

(1) all-ROIs (aROI), the combination of all gray matter voxels within all statistically significant ROIs,
(2) individual ROIs (iROI), the combination of gray matter voxels within a single statistically significant ROI, and
(3) relaxed-criteria all-ROIs (rcaROI {*param*}), i.e., aROIs for which the criteria used to select gray matter voxels for inclusion in the ROIs is relaxed according to the parameter, *param*. We utilized two relaxation methods: 1- and 2-voxel dilation (via AFNI’s 3dmask_tool) was used to expand the sizes of highly significant clusters (*p* ≤ 0.001). We also relaxed the level of uncorrected thresholding we applied prior to cluster size correction, using both *p* ≤ 0.01 and *p* ≤ 0.05 (the minimum cluster sizes in these cases also change: *p* ≤ 0.01 yields a minimum cluster of 43.4 voxels; *p* ≤ 0.05 yields a minimum cluster size of 138.9 voxels).

## Results

### Univariate GLM

The univariate GLM approach yielded a group-level statistical activation map of 14 clusters for the valence contrast (V_pos_ – V_neg_) and 8 clusters for the arousal contrast (A_high_ – A_low_). Surviving regions include many brain areas previously identified within the Neural Reference Space (NRS) (Barrett et al., 2007; Kober et al., 2008), a set of regions identified from neuroanatomical studies and consistently activated across meta-univariate analysis of neuroimaging studies of affective processing. These regions included amygdala, ventrolateral prefrontal cortex (vlPFC), dorsolateral prefrontal cortex (dlPFC), dorsomedial prefrontal cortex (dmPFC), occipital-temporal cortex, and cerebellum (see **Table 1**).

**Table 1:** Summary of brain regions exhibiting significant levels of activation when contrasted between differing stimulus conditions.

Valence contrast activation (pos stim - neg stim)			Arousal contrast activation (high stim - low stim)		
Region (l = left, r = right)	Coord. CoMass x, y, z	Size voxels	Region (l = left, r = right)	Coord. CoMass x, y, z	Size voxels
vlPFC (r)^∗^	41.6, 25.9, 0.9	51	Visual cortex (l)	–52.3, -62.0, 5.3	282
Motor cortex (r)	15.4, -30.6, 68.8	45	Visual cortex (r)	47.2, -68.1, -3.4	231
Motor cortex (l)	–6.1, -25.8, 69.0	44	Parahippocampus (l)	–28.4, -45.8, -8.7	41
Temporal pole (l)^∗^	–51.5, 6.4, -20.6	38	Parahippocampus (r)	25.3, -42.0, -9.4	33
Motor cortex – hand knob (r)	30.8, -29.3, 56.2	29	Precuneus	0.2, -62.0, 32.3	31
dmPFC (l)^∗^	–6.8, 49.5, 32.8	28	Fusiform (l)	–45.0, -44.2, -16.3	22
dlPFC (r)	51.3, 19.6, 23.4	26	amygdala (l)^∗^	–24.1, 2.9, -10.7	20
Inferior parietal (r)	52.4, -52.2, 20.0	22	posterior inferior temporal (l)^∗^	–42.6, -56.6, -22.4	17
Amygdala (l) ^∗^	–25.0, -2.8, -14.6	19			
SMA	2.7, -16.8, 67.5	18			
Angular gyrus (l)^∗^	–59.1, -51.4, 11.7	16			
Cerebellum (l)^∗^	–20.5, -72.1, -36.3	15			
mid CC (r)	5.9, -10.1, 51.1	15			
Precuneus (l)	–14.1, -37.9, 48.4	14			

*Coordinates are reported in Talairach space. ^∗^Indicates part of the Neural Reference Space (Kober et al., 2008).*

**FIGURE 1:**
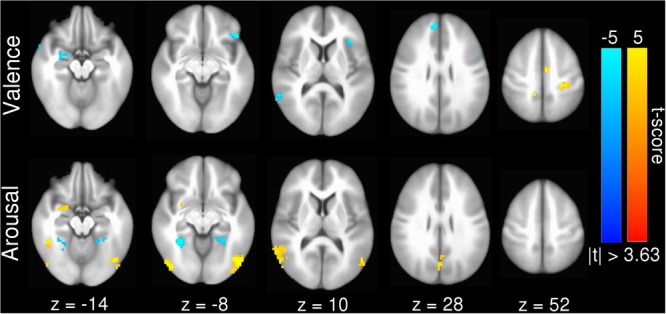
Group statistical map for univariate GLM contrasts of (top) valence (V_pos_ – V_neg_) and (bottom) arousal (A_high_ – A_low_), whole-brain masked. Warm (yellow) colors indicate regions where V_pos_ > V_neg_ or A_high_ > A_low_. Cool (blue) colors indicate regions where V_neg_ > V_poa_ or A_low_ > A_high_. Slices are rendered in axial view using Talairach coordinates and neurological convention (image left = participant left).

Our inter-subject pair-wise contrast activation *t*-scores within the surviving clusters are also consistent with the affective neural processing literature (**Figure 1**). NRS-congruent regions exhibiting greater activation during viewing of negatively valent stimuli include amygdala, temporal pole, dmPFC, and left angular gyrus. Amygdala also showed significant activation in response to high arousal stimuli; no other NRS region showed arousal-related activity.

### Beta-Series Validation

**FIGURE 2:**
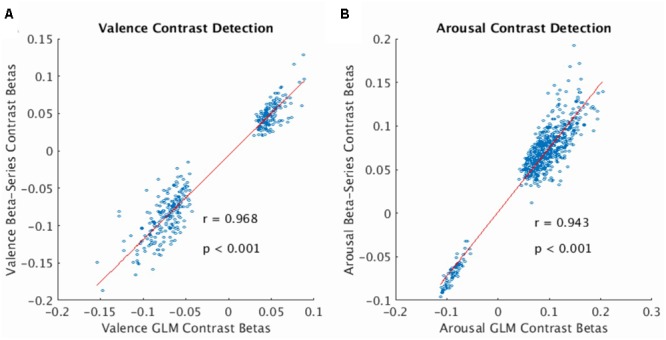
Correlation of voxelwise statistics across univariate and multivariate representations of the BOLD fMRI signal. **(A)** Correlation between mean GLM Contrast Betas and mean Beta-Series Contrast Betas computed for the valence dimension of the stimulus (average calculated over participants). Strong correlation indicates that Beta-Series captures comparable activation information to GLM. **(B)** Same as **(A)** for the arousal dimension of the stimulus. In both plots, red line represents the linear robust regression fit of the data. Comparison voxels were chosen as those voxels simultaneously satisfying the following constraints: group-level GLM contrast betas’ one-sample *t*-statistic having |*t*-score| > 3.63, presence in all 32 folds of the LOOCV of the gray-matter mask (valence voxels = 380; arousal voxels = 628).

The beta-series method produced voxel-wise beta maps which were virtually identical to beta maps derived from univariate GLM contrasts of valence (see **Figure 2A**, Pearson *r* = 0.968, *p* < 0.001) and arousal (see **Figure 2B**, Pearson *r* = 0.943, *p* < 0.001), supporting the use of MPVC to predict the affective dimensions of valence and arousal from multi-voxel patterns of brain activation encoding.

### Cluster Stability Validation

To simplify the ROI-based cluster analysis, we conducted ROI-classification on each participant using a single uniform set of ROI clusters computed from all participants (*n* = 32). To justify this approach, we computed the true cross-validated ROI clusters for each participant. We then mapped these cross-validated clusters onto the group-level clusters to compute the variation in presence, size, and distance of the clusters found. Results of these mappings are presented in **Table 2** and suggest strong stability in the cross-validation clustering. Exceptions in stability were the amygdala, mid-cingulate cortex (mid CC), and angular gyrus ROIs clustered from the valence univariate contrast maps. The amygdala and mid-cingulate cortex exhibited relatively high dropout during cross-validated clustering. Angular gyrus exhibited above average (8.26 mm) cluster center-of-mass variability.

**Table 2:** Inter-subject cross-validated cluster stability analysis.

Cluster Set	Group-Level Cluster, Region (l = left, r = right)	Cluster Fraction Matched	Cluster Size Mean (voxels)	Cluster Size Standard deviation (voxels)	Match Distance Mean (mm)	Match Distance Standard deviation (mm)
Valence	vlPFC (r)	1.00	43.63	6.49	2.22	0.98
	Motor cortex (r)	1.00	46.75	15.00	2.69	2.31
	Motor cortex (l)	1.00	39.28	10.93	1.35	1.92
	Temporal pole (l)	1.00	32.13	4.26	0.64	0.23
	Motor cortex h.k. (r)	0.94	24.67	5.03	0.70	0.61
	dmPFC (l)	1.00	27.66	2.03	0.34	0.26
	dlPFC (r)	1.00	18.68	5.54	1.45	0.65
	Inferior parietal (r)	1.00	20.34	5.00	0.67	0.47
	Amygdala (l)	0.50	22.25	5.21	1.58	1.64
	SMA	0.97	16.68	2.86	0.76	0.62
	Angular gyrus (l)	0.91	21.72	9.94	8.26	10.91
	Cerebellum (l)	0.72	16.04	2.69	0.50	0.55
	mid CC (r)	0.56	16.00	4.64	2.88	8.69
	Precuneus (l)	0.69	14.46	0.91	1.29	4.52
Arousal	Visual cortex (l)	1.00	264.91	22.38	0.75	0.49
	Visual cortex (r)	1.00	202.91	32.52	1.32	0.53
	Parahippocampus (l)	1.00	37.59	5.76	0.54	0.27
	Parahippocampus (r)	1.00	28.34	7.38	1.62	0.81
	Precuneus	0.91	30.31	35.93	9.25	15.79
	Fusiform (l)	0.97	19.55	9.18	0.84	1.16
	Amygdala (l)	0.91	18.76	4.29	0.80	0.36
	post. infr. temp. (l)	0.91	16.10	2.02	0.91	0.70

*Column properties are calculated over the set of matched clusters for each group-level cluster (regions ordered by cluster size as listed in **Table 1**). The Cluster Fraction Matched is calculated as the number of subjects that matched a cross-validated cluster to the specific group-level cluster divided by the total number of subjects, i.e., maximum value equals 1.0. Cluster Size and Match Distance statistics are calculated on the set of matched clusters for the specific group-level cluster.*

### Inter-subject MVPC Prediction Accuracy

**Table 3** reports MVPC performance for inter-subject classification of features based on whole-brain (restricted to cross-validated GM-masked voxels), all ROIs surviving the univariate GLM contrast (aROI), relaxed criteria variants of the aROIs (rcaROI), and individual ROIs (iROI). **Table 3** only reports iROI classification results which were significantly better than chance. A full report of all iROI experiments is provided in Supplementary Table 2. MVPC results may be summarized as follows.

**Table 3:** Inter-subject multivoxel pattern classification summary.

Classification task	fMRI feature (l = left, r = right)	Mean accuracy	Accuracy 95% CI	Mean TPR	Mean FPR
Valence (pos vs. neg)	Whole-brain	0.5893^∗∗^	[0.5684,0.6103]	0.5982	0.4192
	rcaROI {p < 0.05}	0.5535^∗^,^∗∗^	[0.5327,0.5743]		
	rcaROI {2-voxel dilation}	0.5326^∗^	[0.5132,0.5520]		
	aROI	0.5113^∗^	[0.4920,0.5307]		
	iROI: precuneus (l)	0.5240^∗^	[0.5059,0.5421]		
Arousal (high vs. low)	Whole-brain	0.5553^∗∗^	[0.5329,0.5776]	0.5271	0.4165
	aROI {*p* < 0.05}	0.5150	[0.5109,0.5426]		
	aROI {2-voxel dilation}	0.5212	[0.5027,0.5398]		
	aROI	0.5202	[0.5020,0.5384]		
Valence (self-report pos vs. neg)	Whole-brain	0.6103	[0.5949,0.6538]	0.6056	0.3561

*All fMRI features depicted are statistically significantly different (*p* < 0.05) from chance, (two-tailed one-sample *t*-test, null hypothesis = 0.50). TPR = true positive rate. FPR = false positive rate. ^∗^Indicates that the mean of this feature and the whole-brain feature (for the same classification task) are significantly different (*p* < 0.05, two-tailed two-sample *t*-test). ^∗∗^Indicates that the mean of this feature and aROI are significantly different (*p* < 0.05, two-tailed two-sample *t*-test).*

(1) Whole-brain MVPC predictions were significantly more accurate than predictions based on aROI (and all single iROIs), indicating that subthreshold voxels (and potentially ROIs not captured by canonical univariate analysis) play a significant role in affective processing. This result holds even when comparing aROI variants built from very liberal clustering methodology, i.e., *p* ≤ 0.05 significance thresholding and 2-voxel cluster dilation (see Supplementary Figures 1A,B, respectively), which increase the available voxels by more than one order of magnitude (e.g., *p* ≤ 0.05 cluster thresholding of valence contrast betas yielded 5010 voxels, 2-voxel dilation yielded 4523 voxels compared to 380 voxels for the standard *p* ≤ 0.001 significance threshold).
(2) The individual ROI which predicts dimensional affect (valence) significantly better than chance (precuneus) is not a member of the Neural Reference Space.
(3) Whole-brain MVPC prediction of valence was significantly more accurate than prediction of arousal (*p* < 0.05, two-sample *t*-test).
(4) No significant difference existed between MVPC prediction accuracy of normative valence compared with self-reported valence (*p* < 0.05, two-sample *t*-test).

### Intra-subject MVPC Prediction Accuracy

Intra-subject MVPC prediction accuracy was not significantly better than chance (two-tailed one-sample *t*-test, null hypothesis = 0.50) for normative valence measures using whole-brain, aROI, and all but one iROI feature, dmPFC (l). Intra-subject MVPC prediction accuracy was shown to be significantly worse than chance on normative arousal measures using whole-brain and two iROIs: visual cortex (l) and amygdala (l). The remaining features yield prediction accuracies not significantly better than chance (see Supplementary Table 3 for full results).

### Interpreting the SVM Hyperplane in Anatomical Space

**FIGURE 3:**
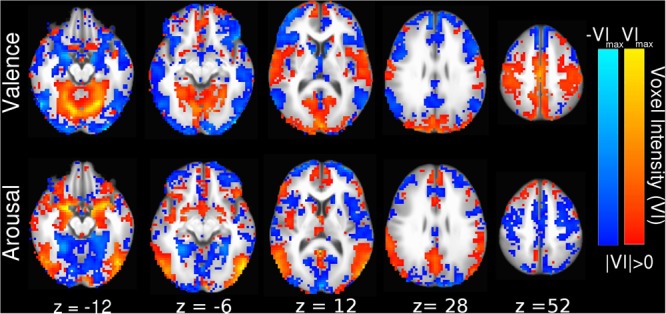
Whole-brain SVM coefficients converted into encoding parameters (Haufe et al., 2014). Colors indicate the strength of activation and the stimulus class under which voxel activation would be observed (red indicating positive valence or high arousal, blue indicating negative valence or low arousal). Slices are depicted in Talairach coordinate space and neurological convention with maximum voxel intensity (VI_max_) = 0.39.

Linear SVM implementation of MVPC allowed us to generate anatomically relevant interpretations of how the brain encodes perceived affect in voxel activation space by converting the SVM decoding coefficients into their equivalent voxel encoding patterns (Haufe et al., 2014; Hebart et al., 2014), which we present in **Figure 3**. The voxel activation patterns learned by our linear SVM also positively correlate with beta-series contrast activations (see Supplementary Figure 2A, valence Pearson correlation *r* = 0.95, *p* < 0.001; see Supplementary Figure 2B, arousal Pearson correlation *r* = 0.87, *p* < 0.001). This indicates that the SVM classifier decodes the voxels’ predominant encoding preference, rather than forming a complex (possibly unnatural and overfit) mapping to maximize prediction performance.

### Interpreting the SVM Hyperplane in Affect Space

SVM hyperplane distance was strongly correlated with normative valence and arousal measures (see Supplementary Figure 3: valence prediction *r* = 0.55, *p* < 0.001; arousal prediction *r* = 0.46, *p* < 0.001). This suggests that MVPA-based regression, via Platt scaling (Platt, 1999), could be used to quantify both the perceived valence and arousal of novel stimuli not previously seen in training in agreement with earlier work supporting the predictive abilities of MVPA in single affect dimensions (Chikazoe et al., 2014; Chang et al., 2015).

**FIGURE 4:**
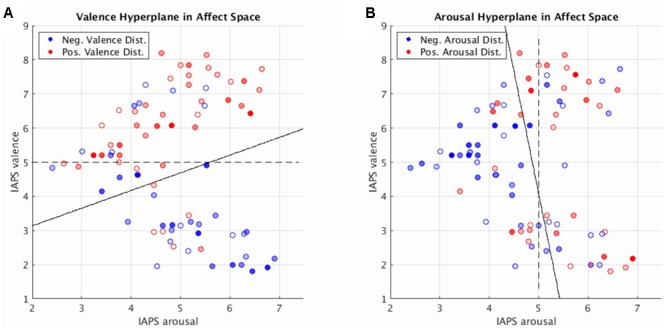
Brain imaging state distances to the linear SVM hyperplane as a function of the normative affect rating coordinates of the stimulus for the prediction of valence **(A)** and arousal **(B)**. Reported distances represent the test set distances averaged over cross-validations. Red hue represents distance in the positive half-plane. Blue hue represents distance in the negative half-plane. Transparency (alpha) represents absolute distance. Full saturation indicates a hyperplane distance ≥ 1.0. Transparent points lie close to the hyperplane (alpha = 0.0 at distance = 0.0). Note, for illustration purposes transparently filled points maintain a full saturation marker edge to indicate their absolute position with respect to the half-plane. Dashed lines represent the theoretically ideal separating hyperplane in 2-dimensional affect space. Solid lines represent the separating hyperplane of a linear SVM in 2-dimensional affect space trained to classify the signs of the hyperplane distances predicted by the brain imaging state SVM hyperplane.

Toward this end, we can elucidate the relationship between dimensional affect ratings and their corresponding brain states by visualizing these states in a low-dimensional representation: mean distance and direction of brain states from the SVM hyperplane as a function of the normative dimensional affect ratings of the stimuli that evoked them. We implemented this approach and display our findings in **Figure 4**. **Figure 4** simultaneously depicts the SVM hyperplane in high-dimensional brain statespace (point colors and transparencies) as well as the theoretically ideal low-dimensional separating hyperplane (dashed line) and the observed low-dimensional hyperplane (solid line) for perceived valence and arousal, respectively. As depicted by **Figure 4**, the observed low-dimensional separating hyperplanes deviate from their theoretical ideals but support the contention that the trained high-dimensional hyperplanes correctly captured the global properties of dimensional affect.

**Figure 4** also illustrates regions within the dimensional affect space for which brain states systematically lay in the wrong half of the high-dimensional hyperplane. These regions of misidentification encompass stimuli with moderate arousal ratings (range of 4–5) with positive or negative normative valence ratings. Such regions of misidentification are evident for both valence and arousal prediction experiments.

**FIGURE 5:**
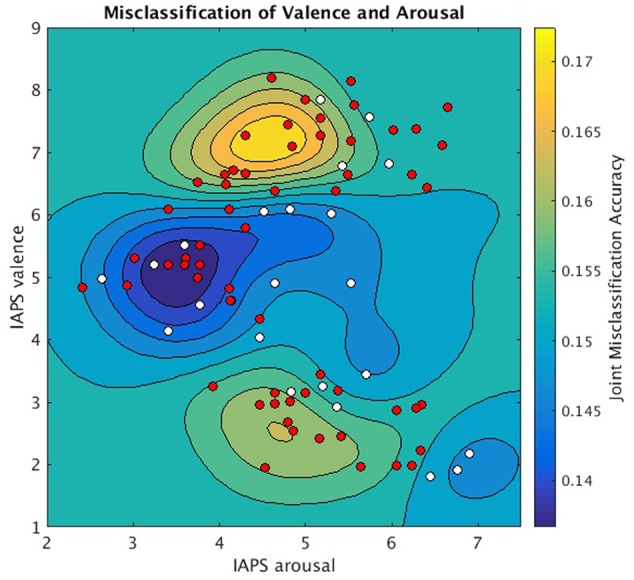
Joint valence and arousal misclassification performance as a function of normative affect space coordinates. Underlay: Gaussian Process Model of mean joint misclassification accuracies assigned to each stimulus in normative affect coordinates. Overlay: stimuli plotted in normative affect space coordinates and labeled according to the significance with which MVPC could jointly classify their valence and arousal labels (one-sample *t*-test, null hypothesis = 0.25, for symbols *p* < 0.05 is white, *p* ≥ 0.05 is red).

Since each image stimulus simultaneously conveys both valence and arousal information, the hyperplanes encoding valence and arousal can be jointly analyzed to model overall prediction accuracy for each stimulus. We implemented this analysis in **Figure 5** to depict the joint misclassification accuracy across both valence and arousal hyperplanes. The contour mapping indicates areas within affect space of similar misclassification accuracy, i.e., reliable prediction (dark blue), and areas of high misclassification accuracy, i.e., unreliable prediction (yellow) as given by a Gaussian Process model of mean joint misclassification accuracy. Greatest misclassification was observed for positively valent stimuli (V∼7.2) associated with slightly below threshold arousal levels (A∼4.5). A secondary misclassification focus was observed for negatively valent stimuli (V∼2.5) also associated with slightly below threshold arousal (A∼4.6).

## Discussion

In this work we used MVPC to map the neural processing predictors of the affective dimensions of valence and arousal. Significant results were observed for inter-subject prediction, wherein a single participant’s brain states reliably predicted the affective dimensions of their observed stimuli from a binary classifier independently trained on datasets pooled from multiple subjects. We reported stronger inter-subject prediction of valence ratings than arousal ratings. Features that incorporated voxels from the whole brain were significantly better predictors of affective dimension ratings than features restricted to specific neuroanatomic regions, supporting a widely distributed pattern of brain activation in rendering such judgments. Furthermore, features using single ROIs corresponding to regions typically associated with affect processing produced significantly worse MVPC-based predictions – often not better than chance. Moreover, our interrogation of the learned inter-subject MVPC hyperplane found that the brain regions canonically associated by univariate approaches with affective processing are indeed relevant.

Combined, these results offer new insights into the neural representations of affective processing. In agreement with a growing body of work based on both univariate (Lindquist et al., 2012; Wager et al., 2015) and multivariate analyses (Baucom et al., 2012; Kassam et al., 2013; Chang et al., 2015; Saarimäki et al., 2016), the attribution of affective processing to any single brain region appears untenable. However, the ability of our multivariate approach to achieve superior inter-subject prediction performance, particularly within a relatively large, normative dataset, suggests some degree of functional stability across individuals of the distributed neural processing underlying affective judgments. This would suggest that, congruent with a recent MVPC-based investigation of discrete emotions (Saarimäki et al., 2016), early stage processing in the dimensional affect model of emotion (Lindquist et al., 2012) can be characterized by distributed, functional homologues of the affective dimensions.

The dissimilarity between anatomical regions implicated in our multivariate analysis and those canonically associated with affective processing in the univariate literature suggests that the stringent clustering threshold employed by univariate analyses to account for multiple comparisons (and thus control Type I error rates) is inflating Type II error by excluding voxels (or possibly ROIs) with significant functional contributions to affective processing. Voxels surviving these univariate clustering approaches constitute functionally relevant ROIs that – as an ensemble (if not independently) constitute the major neural processing correlates of multi-dimensional affective signal processing – but cannot, without the information contained within subthreshold voxels, achieve maximum prediction performance. Our results comparing classifiers built upon ROIs surviving standard (*p* ≤ 0.001) versus relaxed (*p* ≤ 0.05) thresholding support this possibility, which may also explain the contradictory findings among univariate meta-analyses as subthreshold voxels or regions are less likely to be reported in the literature.

However, classifiers built upon whole-brain data significantly outperformed all ROI-based classifiers, even those constructed with highly liberal thresholds, suggesting that voxels outside these theoretically driven ROIs perform crucial affective processing functions, thus confirming and extending earlier MVPC results (Baucom et al., 2012). Collectively, our results support the hypothesis that a distributed model of neural information processing functionally organizes to support the multi-dimensional decoding of affective stimuli, although additional work is necessary to fully understand its anatomical limits beyond the NRS.

A clear implication of this work is the need for caution in the use of single-ROI-based targets for fMRI-based neurofeedback studies of emotion regulation. Although our univariate analyses confirm the amygdala’s recruitment in the processing of images of negative affect and/or high arousal, our MVPC analyses show that the amygdala response alone did not significantly predict these signal encoding properties. Based upon our multivariate analyses, we conclude that fMRI-based neurofeedback may depend on the characterization or classification of a distributed neuromodulation network as a readout to achieve robust volitional emotion regulation.

Our work complements earlier work conducted by Baucom et al. (2012), which used MVPC to classify affective labels from voxel-wise features. Baucom et al.’s (2012) work reported inter-subject peak prediction accuracy (mean accuracy ∼75% for valence and arousal). We attribute this accuracy difference to inter-study differences in stimuli, features, classifier, and participants. Baucom et al. (2012) chose a large number of images (100 per subject) to generate five distinct image sets: a neutral image set (neutral valence, low arousal) and four affectively extreme, highly clustered image sets (positive/negative valence and high/low arousal). Of these, the four extreme image sets (80 images) were used for classification. Conversely, our smaller image set size (45 images per subject) was chosen to span the full continuous range of affective space, offering greater nuance in valence and arousal, which may potentially come at the cost of reduced predictive accuracy in exchange for broader generalizability to everyday stimuli, and may in part explain the recruitment of additional brain regions outside the NRS. Baucom et al. also selected the 2000 most stable voxels across subjects prior to classification, which reduces the dimensionality of the feature space while effectively maintaining the signal to noise ratio, thereby improving MVPC performance. In order to characterize the anatomical encoding of the classification, we conducted classification on the entire set of gray matter voxels. Furthermore, our recruitment practices used broad inclusion criteria and community-based sampling to acquire a more heterogenous sample (in age, ethnicity, and education) than the sample recruited by Baucom et al. (2012) (*n* = 13, 92% female, convenience sampling from a college campus). Although these methodological choices increase feature and label variance that putatively reduce classification performance, we contend that these choices are justified as they provide a more robust understanding of the anatomical constraints of normative affective processing.

A novel finding of our study was that classification of affective images using normative population valence ratings did not significantly differ from classification based upon the study participants own self-reported valence ratings. An implication of this finding is that acquiring each participant’s ratings of affective stimuli may be superfluous for stimulus-based fMRI studies for which norms already exist. Given the expense of MRI scanning and temporal limitations on how long each participant can comfortably tolerate the MRI environment, having participants rate each image may not be prudent – particularly since the rating process may introduce additional performance confounds such as working memory, motor activity, and/or self-referential processes. Instead, having MRI study participants passively view a larger stimulus set would increase the density of samples within the affect space, and thus potentially increase predictive power. The current findings suggest the feasibility of implementing real time fMRI as a means of individually titrating stimulus presentation number based on attainment of a target brain state (Feng et al., 2015). An important caveat, however, is that passive viewing of images may be less attentionally engaging than active rating of images.

We also found specific regions of the affect space that are poorly partitioned by a linear SVM hyperplane. Specifically, regions of extreme positive and negative valence are difficult to classify when combined with moderate arousal. We interpret this regional failure of the hyperplane as the result of a group-level variation in the distribution of brain states corresponding to these stimuli. However, an alternative hypothesis is that sex-based individual variation in our normative sample may contribute to this region of poor classification performance, which is congruent with earlier work (Bradley and Lang, 2007) showing that sex differences drive correlation differences between arousal and valence in the positive valence half-plane. This idea is further bolstered by univariate clusters indicating significant sex differences in pairwise contrast activation (Supplementary Figure 4). Increased mean valence contrast activation was observed in the right vlPFC and right motor cortex of male versus female participants as was increased mean arousal contrast activation observed in the anterior cingulate cortex. These findings suggest that sex differences may impact our ability to characterize both the neural representations of affective valence and arousal in mixed-sex samples. However, no sex differences were observed in whole-brain classification performances for valence (*p* = 0.29, two-sample *t*-test), arousal (*p* = 0.13, two-sample *t*-test), or self-reported valence (*p* = 0.53, two-sample *t*-test). Global neural pattern classification performance may not explain local performance fluctuations. We thus explored sex differences of misclassified stimuli within the regions of highest misclassification shown in **Figure 5**, but found no significant sex differences for valence classification accuracy (*p* = 0.703, two-sample *t*-test) nor arousal (*p* = 0.069, two-sample *t*-test). While these sample sizes are small and may not sufficiently power the comparison, the determinants of these affect space regions of misclassification remains unknown and warrants further investigation.

Our finding of high inter-subject prediction and relatively poor intra-subject prediction accuracy, a result that supports earlier affect state prediction work (Baucom et al., 2012), may reflect anti-learning (Kowalczyk and Chapelle, 2005), a phenomenon in which a classifier systematically learns a high-performing decision hyperplane on the training dataset but prediction performance is worse than chance with LOOCV on an independent test set (see Supplementary Table 3, prediction of normative arousal ratings). Anti-learning is a fundamental property of both synthetic and real-world datasets (Kowalczyk et al., 2007) and has been theoretically linked to data domains exhibiting both high-dimensionality and low sample size (Hall et al., 2005; Kowalczyk and Chapelle, 2005), which typifies neuroimaging studies and is particularly relevant here. Our intra-subject normative arousal predictions were learned from only 40 training examples, each having 36,594 dimensions. In contrast, our inter-subject predictions were learned from 31 times the number of training examples. It follows then that no examples of anti-learning were found during inter-subject prediction.

The strength of our study’s inferences is influenced by clear limitations in experimental approach. The most serious possibility is that the structure of affective processing in the brain’s statespace may be non-linear rather than linear as we assumed. It is possible that observed aROI or iROI results would differ if we had explored other MVPC implementations beyond the linear SVM classifier. Our choice of a linear SVM classifier was primarily made for ease of interpretability rather than to optimize classification performance, as linear classifiers are more intuitively depicted in neuroanatomic space than non-linear classifiers. Thus, our reported iROI results conservatively fall on the lower performance bound of what may be possible using more advanced machine learning techniques.

We report stronger classifier performance for features derived from all univariate ROIs than for single ROIs. It is possible that small combinations of ROIs (pairs, triplets, etc.) would achieve performance comparable to classifiers trained using all ROIs. The difficulty here is that there is no obviously efficient method for optimally selecting samples from the space of possible ROI-networks, having on the order of 14-choose-2 (91) or 14-choose-3 (364) combinations in the case of valence. Local search optimization strategies, when considering the full cost of evaluating a single network configuration over all 32 subjects, are not computationally tractable. Also, the equivalence of small ROI networks to aROI, while more supportive of the canonical neuroanatomical model of affective processing, does not refute the primary conclusion of this work that whole-brain features are superior to ROI-based features as regards classifier performance.

Our use of binary classification may mask the possibility that affective processing naturally aligns with higher-order systems of affect states. The univariate analysis literature provides evidence that brain regions important for affective processing select for either neutral or extreme conditions (Posner et al., 2009). Thus, more accurate classification may be attainable using a ternary state system (e.g., positive, negative, and neutral valence rather than positive and negative, or high, medium, and low arousal rather than high and low). This proof-of-concept analysis lays the foundation for future exploration of multi-state classification of affect encoding.

Moving forward, this work supports a vigorous new look at the use of control theoretic neuroimaging techniques in the study of affective processing: specifically, real-time fMRI (rt-fMRI) methodology for investigating the neural substrates of affect and fMRI-based neurofeedback experiments that employ affect responses to achieve neuromodulation goals. In the first case, our results suggest that rt-fMRI combined with MVPA could form the basis of active learning (Cohn et al., 1996) based neuroimaging studies in which stimulus density varies during experimentation to oversample portions of the affect space that are not well-predicted by the model. In the latter, we propose next generation fMRI-based neuromodulation experiments that utilize model-derived whole-brain read-outs as targets of volitional control to boost emotion regulation.

## Ethics Statement

This study was carried out in accordance with the recommendations of the human research policy of the UAMS Institutional Review Board with written informed consent from all subjects. All subjects gave written informed consent in accordance with the Declaration of Helsinki. The protocol was approved by the UAMS Institutional Review Board.

## Author Contributions

All authors made substantial contributions to this work and agree to be accountable for its integrity and accuracy. KB designed and implemented the analytical experiments. CI and SH designed, implemented, and collected the HCNL affective scoring of images. GJ designed, implemented, and collected the affective task data as well as conducted cluster stability analysis. SH and CK enforced the quality and relevance of the manuscript. KB drafted the manuscript with critical revisions by all contributing authors.

## Conflict of Interest Statement

The authors declare that the research was conducted in the absence of any commercial or financial relationships that could be construed as a potential conflict of interest.
