# Supplementary material for: Distributed Neural Processing Predictors of Multi-dimensional Properties of Affect
Source: Front Hum Neurosci. 2017 Sep 14;11:459. doi: 10.3389/fnhum.2017.00459 (PMC5603694; doi:10.3389/fnhum.2017.00459)
Supplement: Supplementary file 1 [file Table_1.DOCX]

Supplementary Material

**Distributed Neural Processing Predictors of Multi-dimensional Properties of Affective Signals**

Keith A. Bush*, Cory S. Inman, Stephan Hamann, Clinton D. Kilts, G. Andrew James

*** Correspondence:** Keith A. Bush: kabush@uams.edu

# Supplementary Figures and Tables

**Supplementary Table 1.** Image stimuli details.*Normative valence and arousal scores estimated via model fit (see “Affect Label Scaling” in the Main Manuscript).

| **IAPS**  **image**  **ID** | **HCNL**  **image**  **ID** | **ArousalScore** | **Valence**  **Score** |
| --- | --- | --- | --- |
| 2205.jpg  2455.jpg  2590.jpg  2811.jpg  3030.jpg  6360.jpg  6834.jpg  9041.jpg  9417.jpg  9520.jpg  1111.jpg  1271.jpg  6210.JPG  7359.jpg  9031.jpg  9187.jpg  9340.jpg  9561.jpg  9571.jpg  9830.jpg  2155.jpg  2222.jpg  2339.jpg  4640.jpg  5470.jpg  8041.jpg  8080.jpg  8116.jpg  8120.jpg  8180.jpg  8420.JPG  8330.JPG  8190.jpg  8380.JPG  1440.jpg  1463.jpg  1650.jpg  7405.jpg  8206.jpg  2190.JPG  2191.jpg  2240.jpg  2393.jpg  2410.jpg  2484.jpg  2491.jpg  2870.jpg  8121.jpg  8117.jpg  5535.jpg  7185.jpg  7510.jpg  7595.jpg | NHA-32.jpg  NHA-35.jpg  NHB-39.jpg  NHA-41.jpg  NHA-44.jpg  NNA-06.jpg  NNB-09.jpg  NNB-13.jpg  NNA-17.jpg  NNB-42.jpg  PHA-45.jpg  PNA-10.jpg  PNA-18.jpg  PNA-21.jpg  PNA-27.jpg  PNB-28.jpg  PNB-33.jpg  PNB-34.jpg  PNA-37.jpg  PNA-41.jpg  PNB-43.jpg  ZHB-32(b).jpg  ZHA-35.jpg  ZHA-36.jpg  ZHA-41.jpg  ZHB-42.jpg  ZNA-15.jpg  ZNB-18.jpg  ZNB-19.jpg  ZNA-21.jpg  ZNB-24.jpg  ZNB-32.jpg  ZNA-35.jpg  ZNB-40.jpg  ZNA-42.jpg  ZNB-43.jpg  ZNB-44.jpg | 4.530  4.460  3.930  6.900  6.760  6.330  6.280  4.640  4.830  5.410  6.050*  6.229*  6.050*  6.050*  4.99*  5.200  5.370  5.173*  5.701*  5.522*  4.646*  6.340  5.360  4.820  6.450  5.160  4.790  5.640  4.860  4.466*  5.430  4.080  4.160  5.520  6.020  5.490  6.650  5.970  4.850  6.590  5.560  4.060  5.173  5.740  5.173*  4.610  4.790  6.230  5.353*  6.280  5.173*  4.994*  6.410  4.825*  4.297*  4.646*  5.173*  4.118*  4.297*  5.522*  2.410  3.610  3.750  2.930  4.130  3.750  3.410  3.010  4.140  4.297*  5.300  3.769*  3.769*  4.646*  3.769*  4.110  2.640  3.590*  4.520  3.769*  3.590*  3.770  3.410*  3.769*  3.410*  4.118*  4.466*  3.241*  3.590*  3.410* | 1.950  2.960  3.260  2.170  1.910  2.230  2.910  2.980  3.160  2.460  1.985*  1.985*  1.985*  2.865*  3.147*  3.250  3.190  3.446*  3.446*  4.906*  3.147*  2.950  2.920  3.010  1.810  2.410  2.680  1.960  2.540  4.026*  6.780  6.480  6.720  7.180  7.350  6.650  7.730  6.820  7.090  7.120  7.760  6.650  7.264  7.560  7.845*  8.190  7.450  6.650  6.384*  7.380  7.545*  7.845*  6.430  6.085*  7.264*  6.384*  7.264*  6.085*  6.666*  8.144*  4.830  5.300  6.530  4.870  4.620  5.000  4.140  5.310  4.630  5.786*  6.020  5.205*  5.504*  4.906*  5.504*  4.810  4.970  5.205*  6.050  5.205*  5.205*  4.550  5.205*  5.504*  5.205*  6.085*  4.325*  5.205*  5.504*  6.085* |
