# Supplementary material for: Distributed Neural Processing Predictors of Multi-dimensional Properties of Affect
Source: Front Hum Neurosci. 2017 Sep 14;11:459. doi: 10.3389/fnhum.2017.00459 (PMC5603694; doi:10.3389/fnhum.2017.00459)
Supplement: Supplementary file 2 [file Table_2.DOCX]

Supplementary Material

**Distributed Neural Processing Predictors of Multi-dimensional Properties of Affective Signals**

Keith A. Bush*, Cory S. Inman, Stephan Hamann, Clinton D. Kilts, G. Andrew James

*** Correspondence:** Keith A. Bush: kabush@uams.edu

# Supplementary Figures and Tables

**Supplementary Table 2.** Inter-subject multivoxel pattern classification full results. ^+^For ROI features, the reported Mean Accuracy refers to iROI experiments.*Indicates that the mean is significantly different from chance (p<0.05, 1-sample t-test, null hypothesis = 0.50) **Indicates that the mean of this feature and aROI are significantly different (p<0.05, 2-sample t-test). ***Indicates that the mean of this feature and whole-brain are significantly different (p<0.05, 2-sample t-test).

| **Classification**  **Task** | **fMRI Feature**  (l=left, r=right) | **Mean Accuracy^+^** | **Accuracy**  **95% CI** |
| --- | --- | --- | --- |
| Valence  (pos vs neg) | whole-brain | 0.5893*,** | [0.5684,0.6103] |
|  | rcaROI {p<0.05} | 0.5535*,**,*** | [0.5327,0.5743] |
|  | rcaROI {p<0.01} | 0.5267*,*** | [0.5109,0.5426] |
|  | rcaROI {2-voxel dilation} | 0.5326*,*** | [0.5132,0.5520] |
|  | rcaROI {1-voxel dilation} | 0.5307*,*** | [0.5114,0.5499] |
|  | aROI | 0.5113*** | [0.4920,0.5307] |
|  | ROI: vlPFC (r) | 0.5212*** | [0.4962,0.5461] |
|  | ROI: motor cortex (r) | 0.5101*** | [0.4925,0.5276] |
|  | ROI: motor cortex (l) | 0.5031*** | [0.4814,0.5248] |
|  | ROI: temporal pole (l) | 0.4976*** | [0.4787,0.5164] |
|  | ROI: motor cortex-hand  knob (r) | 0.5062*** | [0.4852,0.5271] |
|  | ROI: dmPFC (l) | 0.5070*** | [0.4903,0.5237] |
|  | ROI: dlPFC (r) | 0.5124*** | [0.4991,0.5257] |
|  | ROI: inferior parietal (r) | 0.4882*** | [0.4674,0.5090] |
|  | ROI: amygdala (l) | 0.5003*** | [0.4760,0.5247] |
|  | ROI: SMA | 0.4735*** | [0.4458,0.5012] |
|  | ROI: angular gyrus (l) | 0.4907*** | [0.4691,0.5122] |
|  | ROI: cerebellum (l) | 0.5000*** | [0.4841,0.5160] |
|  | ROI: mid CC (r) | 0.4956*** | [0.4770,0.5141] |
|  | ROI: precuneus (l) | 0.5240*,*** | [0.5059,0.5421] |
| Arousal  (high vs low) | whole-brain | 0.5553*,** | [0.5329,0.5776] |
|  | rcaROI {p<0.05} | 0.5150*,*** | [0.5109,0.5426] |
|  | rcaROI {p<0.01} | 0.5171*** | [0.4964,0.5336] |
|  | rcaROI {2-voxel dilation} | 0.5212*,*** | [0.5027,0.5398] |
|  | rcaROI {1-voxel dilation} | 0.5159*,*** | [0.5006,0.5313] |
|  | aROI | 0.5202*,*** | [0.5020,0.5384] |
|  | ROI: visual cortex (l) | 0.5022*** | [0.4850,0.5194] |
|  | ROI: visual cortex (r) | 0.5119*** | [0.4991,0.5246] |
|  | ROI: parahippocampus (l) | 0.5119*** | [0.4873,0.5365] |
|  | ROI: parahippocampus (r) | 0.5135*** | [0.4984,0.5286] |
|  | ROI: precuneus | 0.4940*** | [0.4780,0.5099] |
|  | ROI: fusiform (l) | 0.5087*** | [0.4900,0.5274] |
|  | ROI: amygdala (l) | 0.5038*** | [0.4892,0.5184] |
|  | ROI: posterior infr. temporal (l) | 0.5147*** | [0.4933,0.5361] |
| Valence  (self-report pos vs neg) | whole-brain | 0.6243* | [0.5949,0.6538] |
