# Supplementary material for: Distributed Neural Processing Predictors of Multi-dimensional Properties of Affect
Source: Front Hum Neurosci. 2017 Sep 14;11:459. doi: 10.3389/fnhum.2017.00459 (PMC5603694; doi:10.3389/fnhum.2017.00459)
Supplement: Supplementary file 3 [file Table_3.DOCX]

Supplementary Material

**Distributed Neural Processing Predictors of Multi-dimensional Properties of Affective Signals**

Keith A. Bush*, Cory S. Inman, Stephan Hamann, Clinton D. Kilts, G. Andrew James

*** Correspondence:** Keith A. Bush: kabush@uams.edu

# Supplementary Figures and Tables

**Supplementary Table 3.** Intra-subject multivoxel pattern classification full results. ^+^For ROI features, the reported Mean Accuracy refers to iROI experiments. *Indicates that the mean is significantly different from chance (p<0.05, 1-sample t-test, null hypothesis = 0.50). **Indicates that the mean of this feature and aROI are significantly different (p<0.05, 2-sample t-test). ***Indicates that the mean of this feature and whole-brain are significantly different (p<0.05, 2-sample t-test).

| **Classification**  **Task** | **fMRI Feature**  (l=left, r=right) | **Mean Accuracy^+^** | **Accuracy**  **95% CI** |
| --- | --- | --- | --- |
| Valence  (pos vs neg) | whole-brain | 0.5228 | [0.4895,0.5562] |
|  | aROI | 0.4993 | [0.4689,0.5296] |
|  | ROI: vlPFC (r) | 0.5231 | [0.4858,0.5604] |
|  | ROI: motor cortex (r) | 0.4745* | [0.4515,0.4974] |
|  | ROI: motor cortex (l) | 0.5203 | [0.4918,0.5488] |
|  | ROI: temporal pole (l) | 0.4920 | [0.4698,0.5143] |
|  | ROI: motor cortex-hand knob (r) | 0.4826 | [0.4574,0.5079] |
|  | ROI: dmPFC (l) | 0.5298* | [0.5119,0.5478] |
|  | ROI: dlPFC (r) | 0.5017 | [0.4763,0.5272] |
|  | ROI: inferior parietal (r) | 0.4951 | [0.4713,0.5189] |
|  | ROI: amygdala (l) | 0.4844 | [0.4625,0.5063] |
|  | ROI: SMA | 0.5035 | [0.4709,0.5362] |
|  | ROI: angular gyrus (l) | 0.5076 | [0.4852,0.5300] |
|  | ROI: cerebellum (l) | 0.4782 | [0.4211,0.5354] |
|  | ROI: mid CC (r) | 0.4915 | [0.4611,0.5219] |
|  | ROI: precuneus (l) | 0.4924 | [0.4619,0.5230] |
| Arousal  (high vs low) | whole-brain | 0.4754* | [0.4557,0.4952] |
|  | aROI | 0.4995 | [0.4776,0.5213] |
|  | ROI: visual cortex (l) | 0.4762* | [0.4544,0.4979] |
|  | ROI: visual cortex (r) | 0.4848 | [0.4587,0.5109] |
|  | ROI: parahippocampus (l) | 0.4912 | [0.4634,0.5190] |
|  | ROI: parahippocampus (r) | 0.5000 | [0.4679,0.5321] |
|  | ROI: precuneus | 0.4874 | [0.4665,0.5083] |
|  | ROI: fusiform (l) | 0.5059 | [0.4868,0.5251] |
|  | ROI: amygdala (l) | 0.4611* | [0.4400,0.4822] |
|  | ROI: posterior infr. temporal (l) | 0.4937 | [0.4716,0.5159] |
| Valence  (self-report  pos vs neg) | whole-brain | 0.5282 | [0.4910,0.5654] |
