# Supplementary material for: Distributed Neural Processing Predictors of Multi-dimensional Properties of Affect
Source: Front Hum Neurosci. 2017 Sep 14;11:459. doi: 10.3389/fnhum.2017.00459 (PMC5603694; doi:10.3389/fnhum.2017.00459)
Supplement: Supplementary file 4 [file Data_Sheet_1.DOCX]

Supplementary Material

**Distributed Neural Processing Predictors of Multi-dimensional Properties of Affective Signals**

Keith A. Bush*, Cory S. Inman, Stephan Hamann, Clinton D. Kilts, G. Andrew James

*** Correspondence:** Keith A. Bush: kabush@uams.edu

# Supplementary Figures and Tables


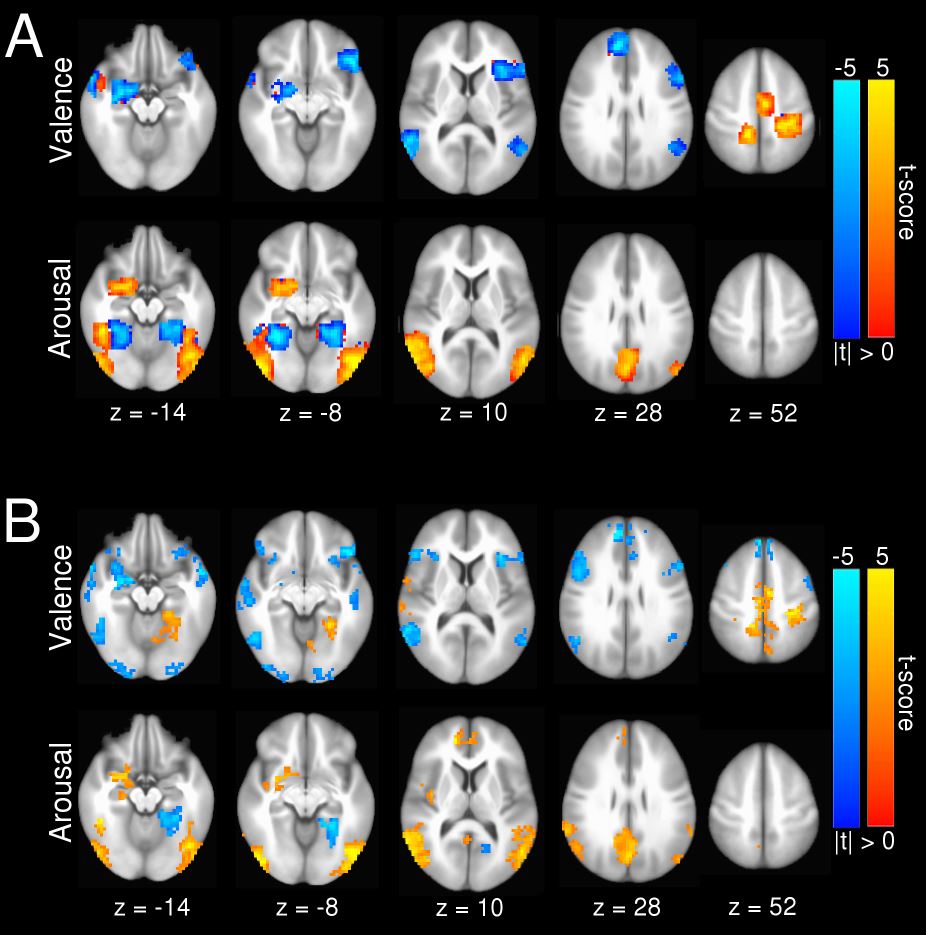


**Supplementary Figure 1.** Group statistical map for univariate GLM contrasts of valence (V_pos_ - V_neg_,) and arousal (A_high_ - A_low_,) for the rcaROI masks. **(A)** rcaROI {2-voxel dilation} mask. **(B)** rcaROI {p≤0.05 threshold} mask Slices are rendered in axial view using Talairach coordinates and neurological convention (image left=participant left).
