# Supplementary material for: Distributed Neural Processing Predictors of Multi-dimensional Properties of Affect
Source: Front Hum Neurosci. 2017 Sep 14;11:459. doi: 10.3389/fnhum.2017.00459 (PMC5603694; doi:10.3389/fnhum.2017.00459)
Supplement: Supplementary file 5 [file Data_Sheet_2.DOCX]

Supplementary Material

**Distributed Neural Processing Predictors of Multi-dimensional Properties of Affective Signals**

Keith A. Bush*, Cory S. Inman, Stephan Hamann, Clinton D. Kilts, G. Andrew James

*** Correspondence:** Keith A. Bush: kabush@uams.edu

# Supplementary Figures and Tables

*
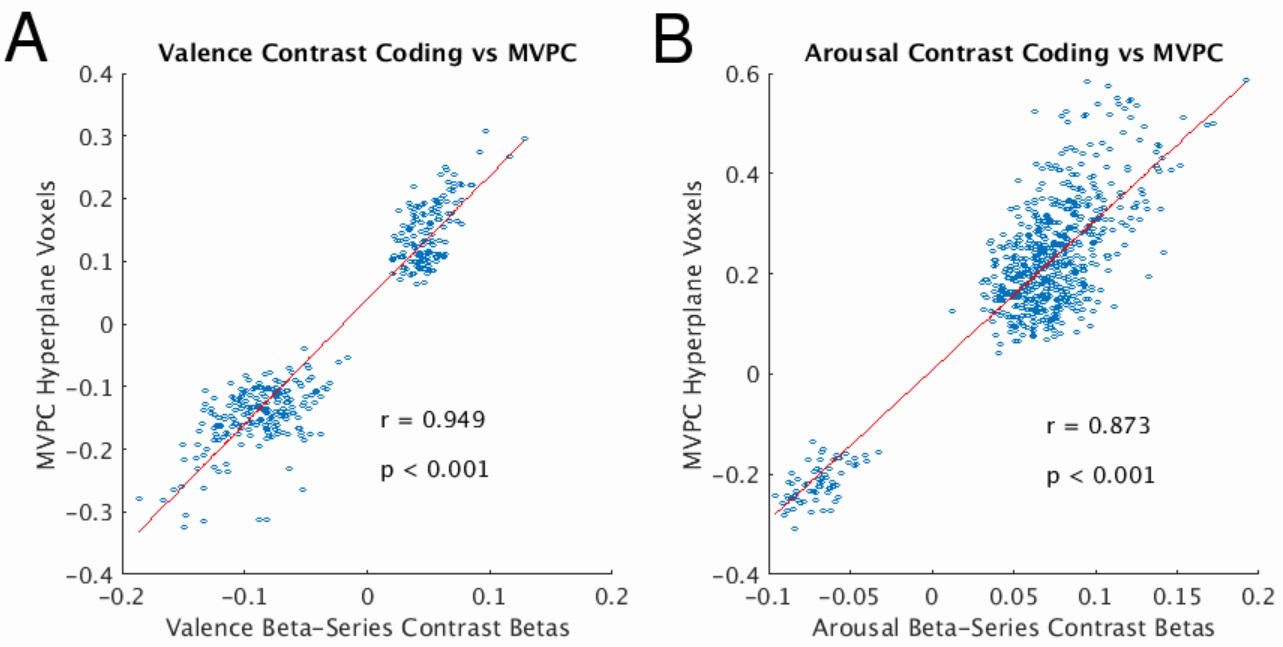
*

**Supplementary Figure 2.** Correlation between mean Beta-Series Contrast Betas and mean cross-validated whole-brain MVPC hyperplane parameters. (**A)** Valence dimension of the stimulus. (**B)** Arousal dimension of the stimulus. Strong correlation indicates that MVPC decodes neural activity in the preferential direction of each voxel, i.e., voxels that activate for a positive stimulus contribute to a positive hyperplane distance and vice versa. In both plots, the red line represents the linear robust regression fit of the data. Comparison voxels (valence voxels = 380; arousal voxels = 628) were chosen as those voxels simultaneously satisfying the following constraints: group-level GLM contrast betas’ one-sample t-statistic having |t-score|>3.63, and presence in all 32 folds of the LOOCV of the gray-matter mask.
