# Supplementary material for: Distributed Neural Processing Predictors of Multi-dimensional Properties of Affect
Source: Front Hum Neurosci. 2017 Sep 14;11:459. doi: 10.3389/fnhum.2017.00459 (PMC5603694; doi:10.3389/fnhum.2017.00459)
Supplement: Supplementary file 6 [file Data_Sheet_3.DOCX]

Supplementary Material

**Distributed Neural Processing Predictors of Multi-dimensional Properties of Affective Signals**

Keith A. Bush*, Cory S. Inman, Stephan Hamann, Clinton D. Kilts, G. Andrew James

*** Correspondence:** Keith A. Bush: kabush@uams.edu

# Supplementary Figures and Tables

*
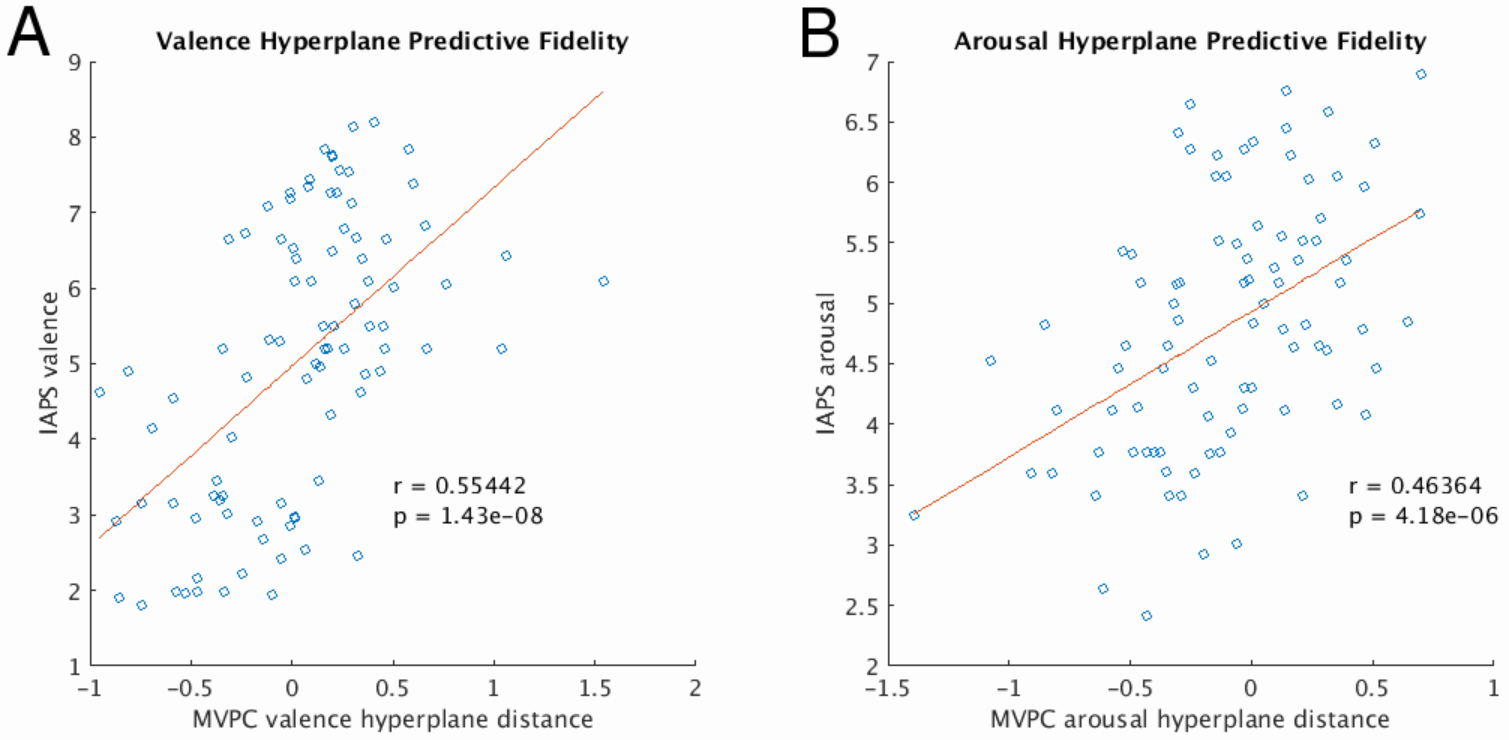
*

**Supplementary Figure 3.** Predictive fidelity of SVM hyperplane distance and direction to normative IAPS Likert Scores across the valence and arousal dimensions. (**A)** SVM hyperplane distance prediction of IAPS valence Likert scores. The high correlation suggests that SVM regression could be used to accurately predict Likert scores based on the BIS response to previously unseen affective stimuli. (**B)** Same as Panel A for the arousal dimension of the stimulus. In all figures the red line represents the linear robust regression fit of the data.
