# Supplementary material for: Distributed Neural Processing Predictors of Multi-dimensional Properties of Affect
Source: Front Hum Neurosci. 2017 Sep 14;11:459. doi: 10.3389/fnhum.2017.00459 (PMC5603694; doi:10.3389/fnhum.2017.00459)
Supplement: Supplementary file 7 [file Data_Sheet_4.DOCX]

Supplementary Material

**Distributed Neural Processing Predictors of Multi-dimensional Properties of Affective Signals**

Keith A. Bush*, Cory S. Inman, Stephan Hamann, Clinton D. Kilts, G. Andrew James

*** Correspondence:** Keith A. Bush: kabush@uams.edu

# Supplementary Figures and Tables


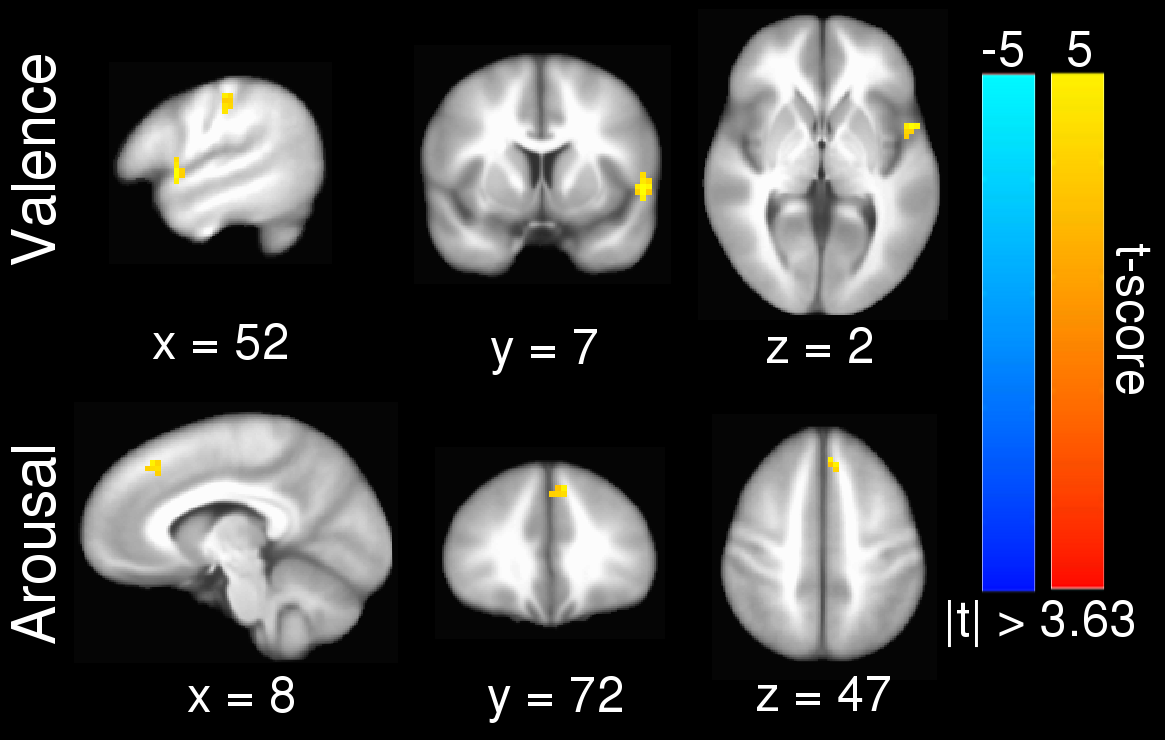


**Supplementary Figure 4.** Gender differences in affective processing. Clusters in the left vlPFC (14 voxels, CoMass: x=52.3, y=6.5, z=2) and motor cortex (18 voxels, CoMass: x=53.4, y=-20.5, z=40.4) depicting significant group-level mean differences (male vs female) of valence contrast activation (V_pos_-V_neg_). A cluster in the anterior cingulate (18 voxels, x=5.1, y=310, z=44.7) depicts significant gender differences of arousal contrast activation (A_high_-A_low_). Positive t-scores signify greater activation for male subjects compared with female subjects. Slices are rendered in axial view using Talairach coordinates and neurological convention (image left=participant left).
